# Development and clinical validation of a novel algorithmic score (GAAD) for detecting HCC in prospective cohort studies

**DOI:** 10.1097/HC9.0000000000000317

**Published:** 2023-11-08

**Authors:** Teerha Piratvisuth, Jinlin Hou, Tawesak Tanwandee, Thomas Berg, Arndt Vogel, Jörg Trojan, Enrico N. De Toni, Masatoshi Kudo, Anja Eiblmaier, Hanns-Georg Klein, Johannes Kolja Hegel, Kairat Madin, Konstantin Kroeniger, Ashish Sharma, Henry L.Y. Chan

**Affiliations:** 1 NKC Institute of Gastroenterology and Hepatology, Songklanagarind Hospital, Prince of Songkla University, Hat Yai, Thailand; 2 Institute of Hepatology, Nanfang Hospital, Southern Medical University, Guangzhou, China; 3 Division of Gastroenterology and Hepatology, Faculty of Medicine Siriraj Hospital, Mahidol University, Bangkok, Thailand; 4 Department of Medicine, Leipzig University Medical Center, Leipzig, Germany; 5 Department of Gastroenterology, Hepatology and Endocrinology, Medical University of Hanover, Hannover, Germany; 6 Goethe University Frankfurt, University Hospital, Medical Clinic 1, Frankfurt, Germany; 7 Department of Medicine II, University Hospital, Ludwig Maximilian University of Munich, Munich, Germany; 8 Department of Gastroenterology and Hepatology Kindai University, Osaka, Japan; 9 Laboratory Services, Microcoat Biotechnologie GmbH, Bernried, Germany; 10 Center of Human Genetics and Laboratory Diagnostics, Munich, Germany; 11 Studies, Collaborations, and Innovation Management, Labor Berlin Charité Vivantes Services GmbH, Berlin, Germany; 12 Roche Diagnostics GmbH, Penzberg, Germany; 13 Clinical Development & Medical Affairs, Roche Diagnostics International AG, Rotkreuz, Switzerland; 14 Faculty of Medicine, The Chinese University of Hong Kong, Hong Kong, China

## Abstract

**Background:**

Alpha-fetoprotein (AFP) and des-gamma carboxyprothrombin (DCP), also known as protein induced by vitamin K absence-II (PIVKA-II [DCP]) are biomarkers for HCC with limited diagnostic value when used in isolation. The novel GAAD algorithm is an *in vitro* diagnostic combining PIVKA-II (DCP) and AFP measurements, age, and gender (biological sex) to generate a semi-quantitative result. We conducted prospective studies to develop, implement, and clinically validate the GAAD algorithm for differentiating HCC (early and all-stage) and benign chronic liver disease (CLD), across disease stages and etiologies.

**Methods:**

Patients aged ≥18 years with HCC or CLD were prospectively enrolled internationally into algorithm development [n = 1084; 309 HCC cases (40.7% early-stage) and 736 controls] and clinical validation studies [n = 877; 366 HCC cases (47.6% early-stage) and 303 controls]. Serum samples were analyzed on a cobas^®^ e 601 analyzer. Performance was assessed using receiver operating characteristic curve analyses to calculate AUC.

**Results:**

For algorithm development, AUC for differentiation between early-stage HCC and CLD was 90.7%, 84.4%, and 77.2% for GAAD, AFP, and PIVKA-II, respectively. The sensitivity of GAAD for the detection of early-stage HCC was 71.8% with 90.0% specificity. Similar results were shown in the clinical validation study; AUC for differentiation between early-stage HCC and CLD was 91.4% with 70.1% sensitivity and 93.7% specificity. GAAD also showed strong specificity, with a lower rate of false positives regardless of disease stage, etiology, or region.

**Conclusions:**

The GAAD algorithm significantly improves early-stage HCC detection for patients with CLD undergoing HCC surveillance. Further phase III and IV studies are warranted to assess the utility of incorporating the algorithm into clinical practice.

## Graphical abstract

**Figure:**
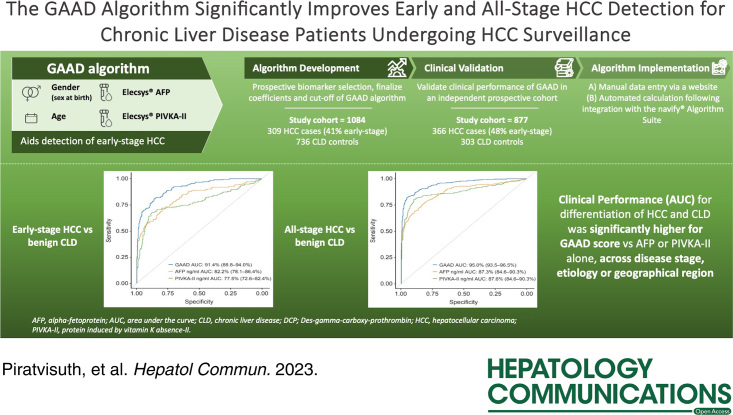

## INTRODUCTION

Liver cancer is the sixth most common cancer worldwide, accounting for ~4.7% of all cancer cases and 8.3% of all cancer deaths in 2020.^1^ HCC represents up to 90% of primary liver cancer cases and most often occurs against a background of chronic liver disease (CLD), such as HBV or HCV infection, NASH, or NAFLD.^2^ Indeed, the cirrhosis resulting from such conditions represents the strongest known risk factor for HCC development.^2,3^

Early-stage HCC is usually asymptomatic. In some regions, it has been estimated that up to 50% of HCC diagnoses are incidental, resulting from the patient presenting with symptoms such as abdominal pain, unexplained weight loss, or liver dysfunction.^2^ Symptomatic HCC is indicative of advanced disease, with diagnosis at this stage generally carrying a poor prognosis, as patients are no longer candidates for transplantation or surgical resection and have limited treatment options.^4,5^ Thus, early HCC diagnosis is essential to improve the prognosis and survival of patients.

Due to the strong association between cirrhosis and the development of HCC, international guidelines recommend surveillance programs involving the screening of at-risk patients, usually with ultrasound scans every 6 months.^5–10^ Despite these recommendations, surveillance and screening programs are not universally implemented and have limited sensitivity in detecting early-stage tumors.^11,12^ Indeed, a meta-analysis characterizing the sensitivity of surveillance imaging found that ultrasound alone was associated with a sensitivity of only 45% for early-stage HCC diagnosis.^13^ Other imaging modalities, such as contrast tomography and MRI, have demonstrated improved sensitivity for HCC; however, their cost and use of contrast agents and/or radiation mean they are infrequently used in surveillance.^14^

To complement surveillance through imaging scans, alpha-fetoprotein (AFP) and des-gamma carboxy-prothrombin, also known as protein induced by vitamin K absence-II (PIVKA-II), have been identified as serum biomarkers that may support in the diagnosis of HCC.^5,15^ Studies demonstrated that patients with HCC had significantly higher PIVKA-II levels compared with healthy controls and patients with benign liver disease.^16^ Moreover, although the sensitivity of AFP alone for HCC detection is limited,^17^ AFP monitoring combined with abdominal ultrasound has been shown to increase the sensitivity for detection of early-stage HCC to 63% compared with 45% with ultrasound alone.^13^

While the diagnostic value of each biomarker in isolation has been the subject of much scientific debate, combining AFP and PIVKA-II has been shown to improve diagnostic efficiency versus each biomarker alone.^18^ Previously, the serum biomarker-based GALAD model was developed to predict the probability of having HCC in patients with CLD. The model accommodates the clinical variables of gender (biological sex) and age alongside 3 serological tumor markers: AFP isoform L3 (AFP-L3), AFP, and PIVKA-II. It has shown utility in supporting diagnostic imaging techniques by demonstrating efficacy in detecting HCC, irrespective of disease stage.^19–21^ However, one study suggested that the presence of the AFP-L3 variable in the GALAD algorithm may have a negligible contribution as the OR for AFP-L3 barely exceeded 1 in both the discovery and validation datasets.^19^

The gender (biological sex), age, AFP, des-gamma carboxy-prothrombin (GAAD) algorithm (Roche Diagnostics International Ltd, Rotkreuz, Switzerland) is a novel *in vitro* multivariate diagnostic, which provides a semi-quantitative result based on gender (biological sex) and age, and 2 serological tumor markers, AFP and PIVKA-II. The biomarkers used in the GAAD algorithm were chosen based on the results of a study that assessed over 50 biomarkers.^22^ Per univariate and multivariate analysis, where an exhaustive search in a nested cross-validation set-up was applied to test all possible marker combinations, PIVKA-II and AFP demonstrated the best individual and combination performance for the identification of both all‐stage and early-stage HCC versus controls.^22^ The ability of the GAAD algorithm to effectively differentiate between HCC and CLD has been demonstrated.^22^ Additionally, comparative analysis versus the GALAD algorithm showed similar performance across disease stages and etiologies.^23,24^

To improve early-stage detection of HCC, we conducted algorithm development and clinical validation studies of the GAAD algorithmic score. The objective of the current study was to establish the GAAD algorithm cutoff values and to validate the clinical performance of the algorithm in differentiating HCC and benign CLD across disease stages and etiologies.

## EXPERIMENTAL PROCEDURES

### Participant selection

Participants were enrolled into one of two independent prospective cohort studies for sample collection: (1) the algorithm training study, which recruited patients aged ≥18 years at seven clinics in Germany, Spain, Thailand, and Hong Kong between 2014 and 2016, and (2) the clinical validation study which recruited patients aged ≥18 years at ten clinics in People’s Republic of China, Germany, Thailand, Hong Kong, and Japan between 2017 and 2022. Each cohort was further divided into an HCC case group or a disease control group, according to national clinical guidelines. The study methodology and prospective trial design for the algorithm development cohort have been described.^22^

The HCC group included patients with a first-time diagnosis of HCC that had been confirmed radiologically (in accordance with international guidelines^5,7,23^) or by liver biopsy within 6 months of enrollment. HCC disease stage was scored using the Barcelona Clinic Liver Cancer (BCLC) staging system, with stages 0–A defined as early-stage HCC and B–D defined as late-stage HCC. The disease control group had a clinical diagnosis of any-etiology cirrhosis or non-cirrhosis (chronic HBV or HCV infection, alcohol-associated liver disease or NASH) and had documented the absence of HCC confirmed by imaging results within the 12 months before enrollment. Those presenting with an indeterminate hepatic mass or a hepatic mass meeting the radiological criteria for HCC were not eligible for inclusion in the control group. Other exclusion criteria across both patient groups included any other form of cancer (excluding non-melanoma skin cancer), recurrent HCC, or previous or current treatment for HCC. Patients were also excluded if they had a glomerular filtration rate < 60 mL/min/1.73 m^2^ or if they were receiving anti-vitamin K coagulant therapy (eg, warfarin, phenprocoumon or acenocoumarol).

All study sites involved in sample collection underwent ethics committee and institutional review board approvals as part of this study, and informed consent was obtained from each participant before enrollment. Local rules regarding informed consent for subsequent use of collected samples were respected.

### Serum sample workflow

Serum samples were collected through patient blood draw ≥ 1 day before any planned procedures involving the administration of general anesthesia. Samples were stored at −70°C at the collecting facilities before being shipped on dry ice to Roche Diagnostics, Penzberg, and then on to the testing site, Microcoat GmbH (Bernried, Germany). Samples collected in the People’s Republic of China were tested at the sample collection site. Good Laboratory Practices and national regulations for shipping of samples were followed throughout. The analysis was conducted over 3 experimental runs using the Elecsys^®^ PIVKA-II and AFP assays on a cobas^®^ e 601 analyzer.

### Clinical performance of the GAAD algorithm

**FIGURE 1:**
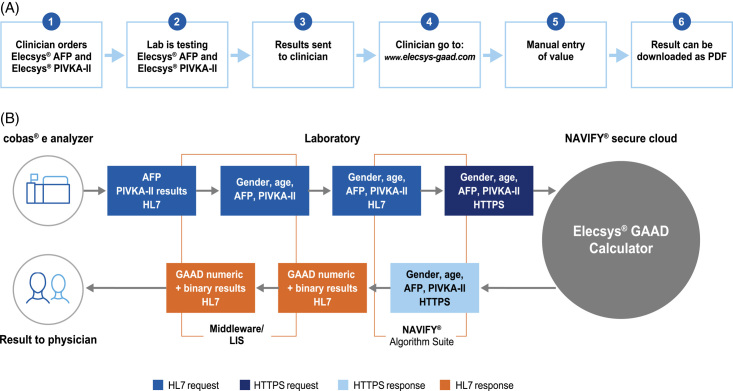
Workflow options with the GAAD algorithm include (A) manual data entry through a website (eService) and (B) automated calculation following integration with the NAVIFY Algorithm Suite. NAVIFY Algorithm Suite automatically pulls clinical and biomarker data from the LIS and calculates the GAAD score to reduce the possibility of manual errors. Abbreviations: AFP, alpha-fetoprotein; DCP, des-gamma carboxy-prothrombin; GAAD, gender (biological sex), age, AFP, DCP; LIS, Laboratory Information System; HL7, Health Level 7; HTTPS, Hypertext Transfer Protocol Secure; PIVKA-II, protein induced by vitamin K absence-II.

The GAAD algorithm is a mathematical formula comprising 2 independent workflow options: (1) manual data entry through a website and (2) automated calculation following integration with the NAVIFY^®^ Algorithm suite, which automatically pulls biomarker data from the Laboratory Information System, Hospital Information System, and/or Electronic Medical Record (Figure 1). The clinical performance of the GAAD algorithm was compared with both the Elecsys^®^ AFP and Elecsys PIVKA-II assays. An additional analysis was performed in a subset of patients, comparing the GAAD algorithm with the clinically validated GALAD algorithm and the Elecsys AFP, AFP-L3, and PIVKA-II assays. The data set comprising gender (biological sex), age, PIVKA-II, and AFP values was run through the calculator unit of the GAAD Web tool. All clinical data were documented in an electronic data capture system, subject to informed consent. Performance was assessed using receiver operating characteristic (ROC) curve analyses to calculate AUC.

### Statistical analysis

The primary objective of this study was to demonstrate that the sensitivity of the GAAD algorithm at its established cutoff score of 2.57^24^ was superior to that of the Elecsys AFP assay at a cutoff of 20 ng/mL or PIVKA-II at a cutoff of 28.4 ng/mL, for the detection of early-stage HCC. In the subset analysis, the established cutoffs were 2.47 for the GALAD algorithm and 2.3 ng/mL for AFP-L3. For sensitivity and specificity of the GAAD algorithm versus the Elecsys AFP and PIVKA-II assays, the derived 95% CIs were calculated from the binomial distribution using the Clopper-Pearson method.^25^ The one-sided McNemar test was used for the comparison of sensitivity between methods.^26,27^ To evaluate the risk of selection bias, several simulations were performed to address the gender and age bias between cases and controls for the GAAD score.

## RESULTS

**FIGURE 2:**
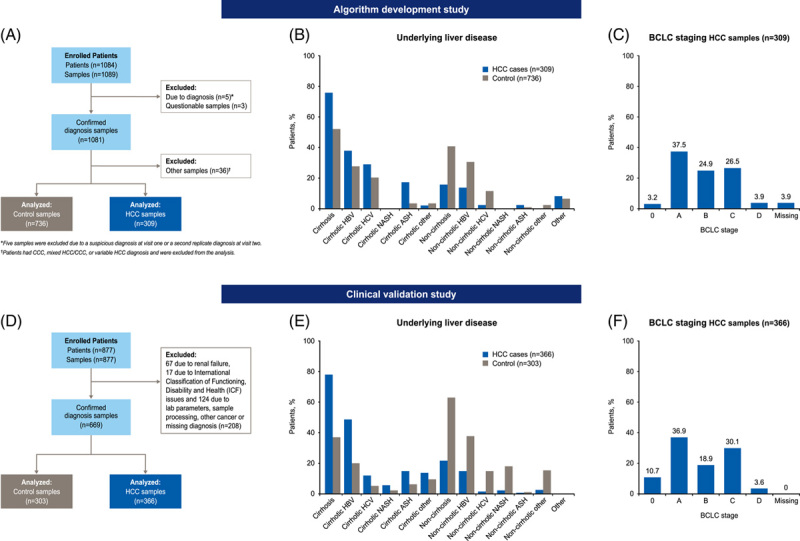
Study disposition, underlying liver disease etiology, and BCLC staging for the algorithm development (A–C) and clinical validation (D–F) cohorts. Abbreviations: ASH, alcohol-associated steatohepatitis; BCLC, Barcelona Clinic Liver Cancer; CCC, cholangiocarcinoma.

In the algorithm development study, 1084 patients were enrolled in 7 centers across Hong Kong, Germany, Spain, and Thailand, with 1089 samples obtained between 2014 and 2016. Eight samples were excluded due to diagnostic errors (n = 5) or as questionable samples (n = 3). A further 36 samples were then excluded as the patients had either cholangiocarcinoma, mixed HCC/ cholangiocarcinoma, or a variable HCC diagnosis. In total, 736 disease control samples and 309 HCC samples were analyzed as part of the algorithm development study (Figure 2A). For the clinical validation study, 877 patients were enrolled in 10 centers across China, Hong Kong, Germany, Japan, and Thailand, with 877 samples obtained between 2017 and 2021. Of these, 208 were excluded (67 due to renal failure, 17 due to International Classification of Functioning, Disability and Health (ICF) issues, and 124 due to lab parameters, sample processing, other cancer, or missing diagnosis). In total, 303 disease control samples and 366 HCC samples were analyzed for clinical validation (Figure 2D).

The baseline characteristics of all patients included in each study are described in Table 1. In both cohorts, most patients were male (Table 1), and the leading etiology was cirrhotic HBV for patients in the HCC group compared with noncirrhotic HBV in the disease control group (Figure 2B and E). In the algorithm development and clinical development cohorts, respectively, 40.7% and 47.6% of patients had very early or early-stage HCC (BCLC Stage 0 or A), while 30.4% and 33.7% had advanced stage HCC (BCLC Stage C or D) (Figure 2C and F). Across both cohorts, mean AST and total bilirubin concentrations were higher in patients with HCC versus controls (Table 1). Tumor characteristics are summarized in Table 2. Baseline characteristics according to region and city of enrollment are provided in Supplemental Table 1, http://links.lww.com/HC9/A643.

**TABLE 1:** Patient characteristics in the algorithm development cohort and clinical validation cohort

	Algorithm development cohort		Clinical validation cohort	
Patient characteristic	HCC (n = 309)	Disease control (n = 736)	HCC (n = 366)	Disease control (n = 303)
Age, y, mean (SD)	60.8 (10.0)	55.5 (10.6)	59.8 (11.4)	49.5 (12.5)
Gender, n (%)				
Male	243 (78.6)	414 (56.3)	308 (84.2)	192 (63.4)
Female	65 (21.0)	320 (43.5)	58 (15.8)	111 (36.6)
Missing	1 (0.3)	2 (0.3)	0	0
Race, n (%)				
Asian	248 (80.3)	637 (86.6)	226 (61.7)	181 (59.7)
White/Caucasian	55 (17.8)	89 (12.1)	138 (37.7)	113 (37.3)
African Black	0	2 (0.3)	1 (0.3)	3 (1.0)
Other	6 (1.9)	8 (1.1)	0	1 (0.3)
Missing	0	0	1 (0.3)	5 (1.7)
Cirrhosis, n (%)	239 (77.3)	395 (53.6)	287 (78.4)	112 (37.0)
Viral liver disease etiology, n (%)	259 (83.8)	670 (91.0)	282 (77.0)	236 (77.9)
Ongoing anti-viral therapy, n (%)				
Yes	81 (26.2)	254 (34.5)	140 (38.3)	121 (39.9)
No	228 (73.8)	482 (65.5)	226 (61.7)	182 (60.1)
Liver biochemistry and prognostic scores				
AST, U/L, mean (SD)	86.9 (85.3)^a^	43.6 (43.3)^b^	72.7 (80.4)	41.6 (99.9)
ALT, U/L, mean (SD)	42.7 (41.0)^a^	31.4 (38.6)^b^	49.8 (52.9)	47.9 (155.0)
PT-INR, n (%)				
1	NA	NA	361 (98.6)	0
2	NA	NA	3 (0.8)	0
3	NA	NA	2 (0.5)	0
Missing	NA	NA	0	303 (100)
Ascites, n (%)				
Mild	NA	NA	54 (14.8)	8 (2.6)
Moderate to severe	NA	NA	21 (5.7)	4 (1.3)
None	NA	NA	291 (79.5)	291 (96.0)
HE, n (%)				
Grade I-II	NA	NA	6 (1.6)	4 (1.3)
None	NA	NA	360 (98.4)	299 (98.7)
Serum albumin, g/L mean (SD)	37.3 (8.8)^c^	42.6 (6.0)^d^	37.1 (5.7)	45.6 (32.7)
Serum total bilirubin, µmol/L, mean (SD)	30.0 (49.0)^e^	18.0 (32.5)^f^	22.2 (26.7)	16.8 (30.0)
MELD score, mean (SD)	NA	NA	8.7 (2.8)	7.4 (1.7)
ALBI score, mean (SD)	−2.3 (0.85)^g^	−2.9 (0.6)^h^	−2.4 (0.59)	−3.2 (2.79)
ALBI grade, n (%)				
I	117 (37.9)	439 (59.7)	140 (38.3)	241 (79.5)
II	134 (43.4)	102 (13.9)	199 (54.4)	56 (18.5)
III	33 (10.7)	16 (2.2)	27 (7.4)	6 (2.0)
Missing	25 (8.1)	179 (24.3)	0	0

a N = 302.

b N = 711.

c N = 289.

d N = 612.

e N = 288.

f N = 565.

g N = 284.

h N = 557.

Abbreviations: ALBI, albumin-bilirubin; PT-INR, prothrombin time-international normalized ratio.

**TABLE 2:** Tumor characteristics for patients with HCC in the algorithm development cohort and clinical validation cohort

Tumor characteristic	Algorithm development cohort (n = 309)	Clinical validation cohort (n = 366)
Performance status, n (%)		
0	NA	270 (73.8)
1	NA	80 (21.9)
2	NA	12 (3.3)
3	NA	3 (0.8)
4	NA	1 (0.3)
Child-Pugh class, n (%)		
A	221 (71.5)	283 (77.3)
B	60 (19.4)	74 (20.2)
C	10 (3.2)	9 (2.5)
Unknown	18 (5.8)	0
Tumor number		
1	0	192 (52.5)
2	62 (20.1)	58 (15.8)
≥3	54 (17.5)	116 (31.7)
Missing	193 (62.5)	0 (0)
Tumor characteristics, n (%)		
Large multinodular	104 (33.7)	87 (23.8)
Portal invasion or extrahepatic spread (N1, M1)	NA	82 (22.4)
Single <2 cm	34 (11.0)	43 (11.7)
Single or ≤3 nodules ≤3 cm	155 (50.2)	154 (42.1)
Size of index lesion, cm, median (IQR; minimum, maximum)	NA	4 (2.5–7.5; 0–18.4)^a^

a N=359.

### Algorithm development

The GAAD score is a logistic regression model, which is trained on all HCC cases and controls from the Algorithm training cohort, with the intended use as an aid in the early diagnosis of HCC. The reason for this is the larger amount of total HCC cases (n = 309) with respect to early HCC cases (n = 126), which yields a model that is more robust based on cross-validated performance estimates. Using the derived coefficients, cutoffs are determined for GAAD to differentiate between early-stage HCC and benign liver disease controls.

**FIGURE 3:**
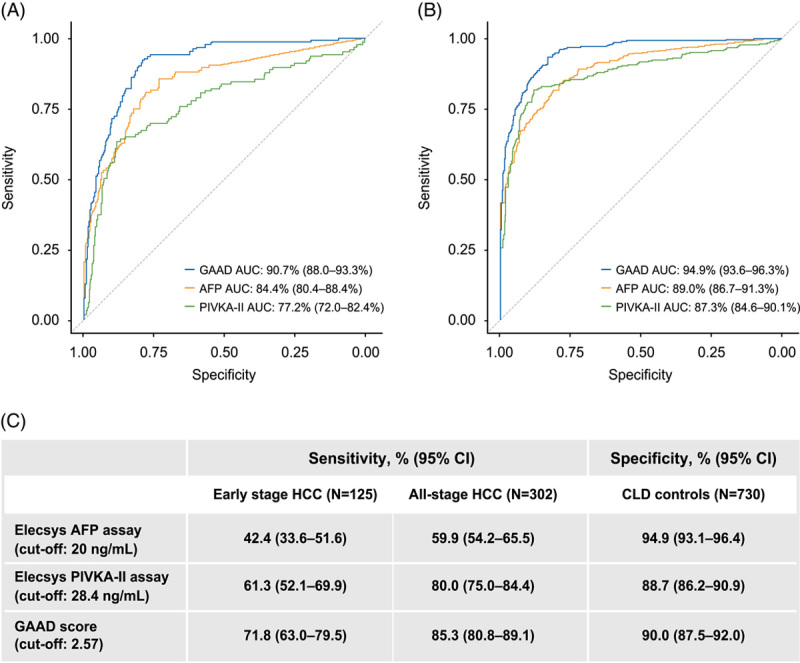
Clinical performance of GAAD versus Elecsys AFP versus Elecsys PIVKA-II in the algorithm development cohort for detection of (A) early-stage HCC versus benign disease control and (B) all-stage HCC versus benign disease control. Sensitivities and specificities are shown in (C). Abbreviations: AFP, alpha-fetoprotein; DCP, des-gamma carboxy-prothrombin; GAAD, Gender (biological sex), Age, AFP, DCP; PIVKA-II, protein induced by vitamin K absence-II.

ROC curves were generated to visualize the sensitivity of the GAAD algorithm in the detection of early-stage and all-stage HCC. For the differentiation of early-stage HCC from benign CLD in the algorithm development cohort, the AUC was highest with GAAD versus AFP or PIVKA-II alone (90.7% vs. 84.4% vs. 77.2%, respectively, Figure 3A). Similar performance was seen with the differentiation of all-stage HCC from benign CLD, again with GAAD showing the highest AUC versus AFP or PIVKA-II (94.9% vs. 89% vs. 87.3%, respectively, Figure 3B). Figure 3C shows the performance of the GAAD algorithm in the algorithm development cohort, where sensitivity for all-stage HCC was 85.3% compared with 71.8% for early-stage HCC, at a specificity of 90% for both stages.

### Clinical validation

**FIGURE 4:**
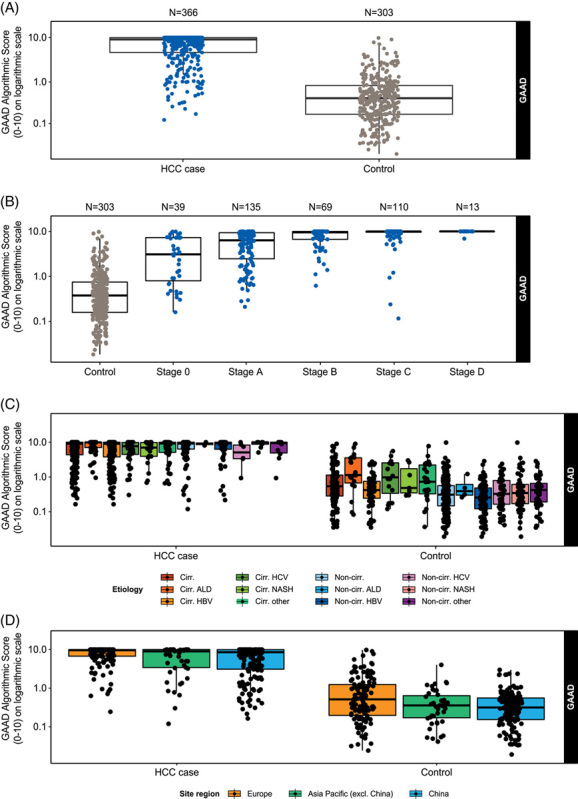
Distribution of GAAD score in HCC cases and benign disease controls in (A) the overall clinical validation cohort; (B) by HCC BCLC stage; (C) by disease etiology; (D) by geographical region. Abbreviations: ALD, alcohol-associated liver disease; BCLC, Barcelona Clinic Liver Cancer; DCP, des-gamma carboxy-prothrombin; GAAD, Gender (biological sex), Age, AFP, DCP.

To examine the distribution of GAAD scores across HCC cases and controls according to disease stage, region and disease etiology, scores were visualized on box plots (Figure 4). A logarithmic axis was selected to present the broad range of scores observed most efficiently.

Within the clinical validation study, the distribution of GAAD scores was notably higher in HCC cases compared with disease controls (Figure 4A): in the HCC group, the median GAAD score was 9.05 (IQR, 4.49–9.96), compared with 0.385 (IQR, 0.162–0.768) in the disease control group. The highest GAAD scores were seen in patients with more advanced HCC. Examining the distribution of GAAD scores according to BCLC stage (Figure 4B), the median GAAD score ranged from 3.1 (IQR, 0.814–7.28) in patients with BCLC stage 0 HCC to 10 (IQR, not evaluated) in patients with BCLC stage D HCC (although only 13 patients were recorded at this late disease stage). GAAD score ranges according to HCC disease stage are described in Supplemental Figure 1, http://links.lww.com/HC9/A643.

Within the HCC case group, GAAD scores remained relatively high, irrespective of the underlying disease etiology (Figure 4C). By comparison, median GAAD scores according to etiology were more variable in the disease control group, ranging from 0.259 (IQR, 0.121–0.488) for noncirrhotic HBV to 1.13 (IQR, not evaluated) with cirrhotic alcohol-associated liver disease. The geographical region seemed to have a minimal impact on GAAD score within the different groups (Figure 4D). Value distributions for the Elecsys AFP and Elecsys PIVKA-II assays are included in Supplemental Figures 2, http://links.lww.com/HC9/A643 and 3, http://links.lww.com/HC9/A643.

**FIGURE 5:**
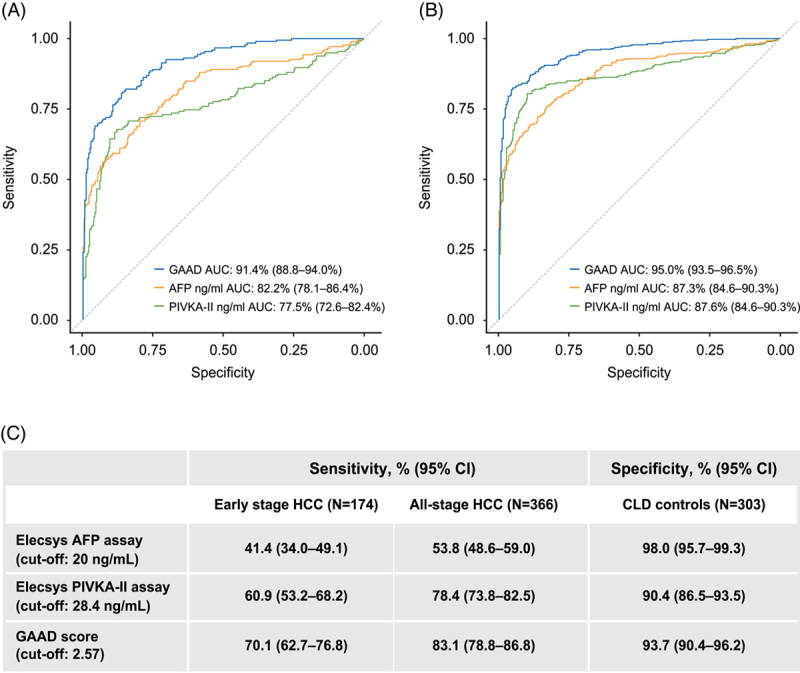
Clinical performance of GAAD versus Elecsys AFP versus Elecsys PIVKA-II in the clinical validation cohort, for detection of (A) early-stage HCC versus benign disease control and (B) all-stage HCC versus benign disease control. Sensitivities and specificities are shown in (C). Abbreviations: AFP, alpha-fetoprotein; CLD, chronic liver disease; DCP, des-gamma carboxy-prothrombin; GAAD, Gender (biological sex), Age, AFP, DCP; PIVKA-II, protein induced by vitamin K absence-II.

Data from the clinical validation cohort were similar to those generated in the algorithm development cohort, showing high AUC with GAAD for differentiation of both early-stage HCC [91.4% (95% CI: 88.8–94.0%)] and all-stage HCC [95.0% (95% CI: 93.5–96.5%)] from benign CLD (Figure 5A and B).

Considering pre-defined cutoffs, GAAD had the highest rate of true positive detection across early-stage, late-stage and all-stage HCC, demonstrating higher sensitivity versus both the AFP and PIVKA-II assays (Supplemental Table 2, http://links.lww.com/HC9/A643; Supplemental Table 3, http://links.lww.com/HC9/A643). In the clinical validation cohort, for early-stage HCC, GAAD correctly detected 122 out of 174 cases [70.1% sensitivity (95% CI: 62.7–76.8)], while AFP alone detected 72 out of 174 cases [41.4% sensitivity (95% CI: 34.0–49.1)] and PIVKA-II alone detected 106 out of 174 cases [60.9% sensitivity (95% CI: 53.2–68.2)]. Sensitivity was higher for all-stage HCC across all tests: GAAD correctly detected 304 out of 366 cases [83.1% sensitivity (95% CI: 78.8–86.8)], while AFP alone detected 197 out of 366 cases [53.8% sensitivity (95% CI: 48.6–59.0)] and PIVKA-II alone detected 287 out of 366 cases [78.4% sensitivity (95% CI: 73.8–82.5)]. Venn diagrams showing the number of early-stage, late-stage and all-stage HCC cases detected by AFP, PIVKA-II, and GAAD found that GAAD detected 8 more HCC cases (early and late), which were missed by the AFP + PIVKA-II combination (Supplemental Figure 4, http://links.lww.com/HC9/A643).

GAAD also showed strong specificity, with high rates of true negative detection regardless of disease stage. However, specificity was higher with the AFP assay (Supplemental Table 2, http://links.lww.com/HC9/A643; Supplemental Table 4, http://links.lww.com/HC9/A643). In the clinical validation cohort, GAAD correctly differentiated 284 of 303 benign disease control samples from early-stage HCC [specificity: 93.7% (95% CI: 90.4–96.2)], compared with 297 of 303 for AFP [specificity: 98.0% (95% CI: 95.7–99.3)] and 274 of 303 for PIVKA-II [specificity: 90.4% (95% CI: 86.5–93.5)]. The same results were also seen when differentiating benign disease control samples from early-stage and all-stage HCC (Supplemental Table 2, http://links.lww.com/HC9/A643). The clinical performance of AFP, PIVKA-II, and GAAD for detection of early-stage, late-stage, and all-stage HCC at the pre-defined cutoffs are shown in Supplemental Table 5, http://links.lww.com/HC9/A643. Several simulations were performed to address the gender (biological sex) and age bias between cases and controls for the GAAD score. When correcting for the differences in age distributions, the performance measures of the GAAD score, which were derived in the prospective study, remained stable (Supplemental Figure 5, http://links.lww.com/HC9/A643). However, when correcting for the differences in gender (biological sex), there was a slight decrease in performance for early-stage and all-stage HCC (Supplemental Figure 6, http://links.lww.com/HC9/A643). A total of 465 patients were included in the sub-analysis comparing the clinical performance of GAAD with GALAD, of which there were 246 cases of HCC and 219 CLD controls. Both algorithms demonstrated similar performance for differentiating HCC and CLD (Supplemental Figure 7, http://links.lww.com/HC9/A643).

**FIGURE 6:**
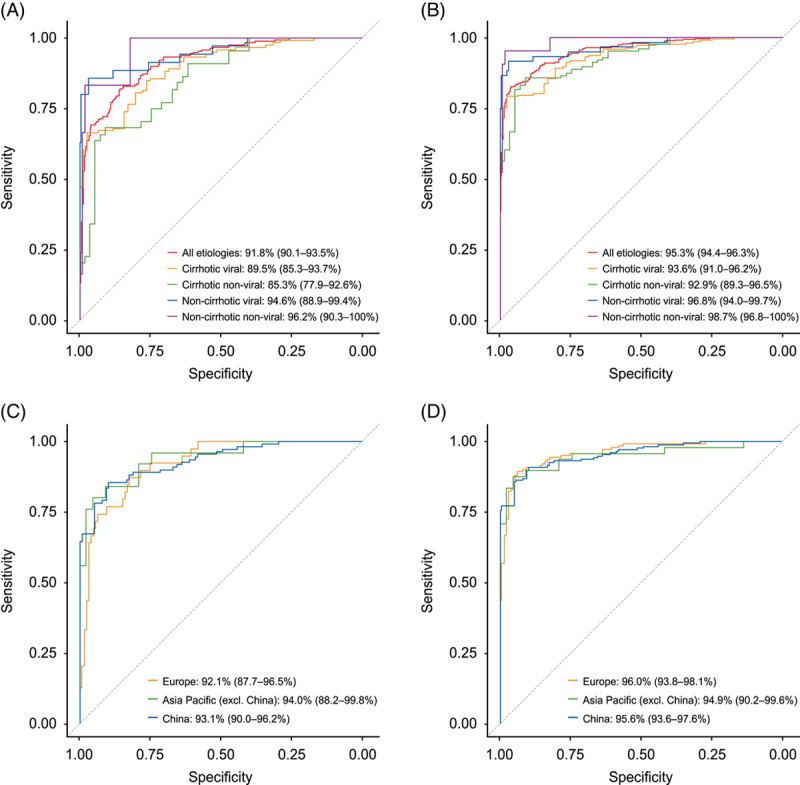
Clinical performance of GAAD for detection of (A) early-stage HCC versus benign disease control according to disease etiology; (B) all-stage HCC versus benign disease control according to disease etiology; (C) early-stage HCC versus benign disease control according to region; (D) all-stage HCC versus benign disease control according to region. Abbreviations: DCP, des-gamma carboxy-prothrombin; GAAD, Gender (biological sex), Age, AFP, DCP.

The GAAD algorithm performed well in differentiating both early-stage and all-stage HCC from benign disease controls across disease etiologies and regions (Figure 6). Considering early-stage HCC, AUC ranged from 85.3% (95% CI: 77.9–92.6) in cirrhotic nonviral disease to 96.2% for noncirrhotic nonviral disease (95% CI: 90.3–100). Similar results were seen for all-stage HCC, where AUC ranged from 92.9% (89.3–96.5) in cirrhotic nonviral disease to 98.7% (95% CI: 96.8–100) in noncirrhotic nonviral disease. The region had minimal impact on AUC, regardless of disease stage. For early-stage HCC, AUC ranged from 92.1% (95% CI: 87.7–96.5) in Europe to 94.0% (95% CI: 88.2–99.8) in Asia-Pacific (excluding China). For differentiation of all-stage HCC, AUC was highest in Europe [96.0% (95% CI: 93.8–98.1)] and lowest in Asia-Pacific [excluding China; 94.9% (95% CI: 90.2–99.6)].

## DISCUSSION

These data support the clinical performance of the GAAD algorithm in the differentiation of HCC from benign CLD. ROC analyses in the algorithm development portion of this study showed higher AUCs for the detection of both early-stage and all-stage HCC with the GAAD algorithm versus the Elecsys AFP and PIVKA-II assays alone. These results were validated in a clinical cohort with GAAD shown to be an effective tool for the differentiation of HCC from disease controls across disease stages, etiologies, and regions.

Within the clinical validation cohort, at a cutoff of 2.57, the GAAD algorithm demonstrated 70.1% sensitivity for the detection of early-stage HCC. This was higher than the sensitivity achieved with both the Elecsys AFP assay (cutoff 20 ng/mL) or the PIVKA-II assay (cutoff 28.4 ng/mL) alone. The GAAD algorithm also demonstrated strong specificity (93.7%), with high rates of true negative detection and low rates of false positives, regardless of disease stage. Combining gender (biological sex) and age with the established biomarkers AFP and PIVKA-II in a single diagnostic algorithm has the potential to improve the differentiation of early-stage HCC from benign CLD versus AFP and PIVKA-II alone. Furthermore, this is achieved without the need for CT scan or MRI referrals. However, while the results of the study are encouraging, phase III and IV studies are needed to fully assess the clinical utility of the GAAD algorithm.

The benefits of regular monitoring for HCC have been known for many years. In 2004, a Chinese study demonstrated that biannual screening with ultrasound and serum AFP could reduce HCC mortality by 37% in a population of 18,816 patients with HBV or a history of chronic hepatitis.^28^ While imaging is still at the foundation of HCC surveillance and diagnosis, and diagnostic algorithms based on serological biomarkers are likely to enhance existing diagnostic procedures rather than replace them, embracing algorithmic analytics may improve the efficiency of such diagnostic workflows. To protect patients from potential harm as a result of HCC surveillance, screening techniques must be as specific and as sensitive as possible. A study in a large cohort of patients with cirrhosis found that over 20% of patients faced “surveillance harms” as a result of false-positive or indeterminate results when relying solely on ultrasound for screening.^29^ Supplementing imaging techniques with serological algorithms to improve diagnostic performance may avoid such harm for patients with CLD and/or HCC. Indeed, combining the GALAD score with ultrasound has been shown to significantly improve the diagnostic performance of the algorithm itself, highlighting the benefit of utilizing both imaging-based and biomarker-based approaches in the diagnosis of early-stage HCC.^30^

The cost-effectiveness of existing screening strategies for HCC has been largely dependent on HCC disease etiology, with not all etiologies reported to be cost-effective.^31^ Therefore, there is a need for cost-effective surveillance techniques, such as GAAD, which are capable of detecting early-stage HCC regardless of HCC disease type. Indeed, one study demonstrated that GAAD was the most cost-effective screening strategy for HCC when compared with ultrasound + AFP in a cohort of UK and Chinese patients.^32^

Optimizing workflows to meet the needs of clinicians and patients is of paramount importance. Automation of tasks can streamline processes and free up resources. For example, a study assessing the economic impact of the NAVIFY multidisciplinary tumor board for decision-making in cancer care found that its implementation provided significant reductions in case preparation and personnel costs through improved workflow efficiencies.^33^

As previously mentioned, the GAAD algorithm is a mathematical formula that implements 2 independent workflow options: (1) website solution with user login and manual data entry and (2) integrated solution through the NAVIFY Algorithm suite (Figure 1). This software solution leverages diverse data as inputs (eg, Laboratory Information System, Electronic Medical Record, etc), applied to a library of clinically useful algorithms founded on a strong evidence base (eg, scientific publications) to deliver outputs that help improve clinical decisions and guide clinical workflows. Furthermore, it has the potential to support structured decision-making to provide clear recommendations or orientation, depending on well-defined and assessable decision criteria integrated within a clinical workflow. Considering the GAAD algorithm alongside the clinically validated GALAD algorithm, GAAD may make reimbursement easier to obtain as it removes the need for AFP-L3 profiling, which could reduce clinical expenses and manual workup.

The GAAD algorithm, combining AFP and PIVKA-II biomarker levels with age and gender (biological sex), was effective in distinguishing between HCC and benign CLD across all disease stages and etiologies assessed. Within the subset analysis comparing the clinical performance of GAAD with GALAD, both algorithms showed good clinical performance for the detection of early-stage and all-stage HCC, with performance maintained across cirrhotic and noncirrhotic etiologies. These data support studies suggesting that the addition of the AFP-L3 assay within the GALAD algorithm has a negligible impact on clinical performance.^19^ The performance of the GAAD and GALAD algorithms will be explored further in a future publication.

For the detection of both early-stage and all-stage HCC, the GAAD score performed better than both the Elecsys AFP and Elecsys PIVKA-II assays alone. Indeed, during the algorithm development study, univariate ROC analysis showed that none of the biomarkers tested met the clinical requirements for early-stage or all-stage HCC detection, with AUC of 83.4% for AFP, 79.0% for PIVKA-II and 76.5% for AFP-L3.^22^

It is important to note the limitations of this study. Although all patients were recruited prospectively, the patients without cirrhosis were analyzed under a different surveillance protocol to the HCC group. We expect that the majority of the patients with noncirrhotic CLD would have carried an indication of HCC surveillance, but unfortunately, our data collection did not capture this level of granularity (eg, Fibrosis stage reporting, etc). It should also be highlighted that the enrolled subjects in the study were not matched for age and gender. To mitigate this, several simulations were performed to address the gender and age bias between cases and controls for the GAAD score. Based on these simulations, the performance of the GAAD score remained stable for both resampling schemes (with or without replacement) and all investigated sample sizes. It is also acknowledged that there are clinical data missing for some patients in the algorithm development cohort. There were a limited number of clinical parameters collected for the algorithm development cohort. However, for the clinical validation cohort, the electronic case report forms had been updated to include more demographic and clinical co-variables.

These findings demonstrate good clinical performance of the GAAD score for the detection of early-stage HCC in patients with CLD who are undergoing regular surveillance. Further study is warranted to assess the utility of incorporating the algorithm into clinical practice.

## Supplementary Material

## Data Availability

Requests concerning the data supporting the findings of this study can be directed to rotkreuz.datasharingrequests@roche.com for consideration.
