# Supplementary material for: Development and clinical validation of a novel algorithmic score (GAAD) for detecting HCC in prospective cohort studies
Source: Hepatol Commun. 2023 Nov 8;7(11):e0317. doi: 10.1097/HC9.0000000000000317 (PMC10635602; doi:10.1097/HC9.0000000000000317)
Supplement: Supplementary file 1 [file hc9-7-e0317-s001.docx]

**Development and Validation of a Novel Algorithmic Score (GAAD) for Detecting Hepatocellular Carcinoma in Prospective Cohort Studies: Supplementary material**

**Supplementary Table 1. Baseline characteristics for participants in the clinical validation cohort according to the clinical site.**

|  | **Europe** | | | | | | **China** | | **Asia-Pacific** | | **Total**  **(N=669)** |
| --- | --- | --- | --- | --- | --- | --- | --- | --- | --- | --- | --- |
|  | **Berlin (N=1)** | **Frankfurt (N=47)** | **Hannover (N=88)** | **Leipzig (N=93)** | **Mainz**  **(N=8)** | **Munich (N=29)** | **Hong Kong (N=109)** | **Guangzhou (N=203)** | **Hat Yai (N=75)** | **Osaka (N=16)** |  |
| Age | | | | | | | | | | | |
| Mean, years | 64 | 64.4 | 55.7 | 59.9 | 51.6 | 58.2 | 58.3 | 47.9 | 54 | 67.9 | 55.1 |
| SD | - | 12.9 | 14.8 | 12.2 | 13.7 | 10.8 | 10.3 | 11 | 10.4 | 12.7 | 13 |
| Median, years | 64 | 64 | 57 | 61 | 54 | 56 | 59 | 48 | 54 | 68.5 | 55 |
| P25–P75, years | 64–64 | 56.5–74 | 45–66.3 | 52–69 | 45–60 | 51–67 | 53–66 | 39.5–55 | 47.5–60.5 | 66.5–74 | 46–65 |
| Min–Max, years | 64–64 | 30–88 | 23–80 | 24–80 | 26–69 | 38–80 | 32–82 | 21–77 | 31–81 | 34–87 | 21–88 |
| Missing,  n (%) | 0 (0) | 0 (0) | 0 (0) | 0 (0) | 0 (0) | 0 (0) | 0 (0) | 0 (0) | 0 (0) | 0 (0) | 0 (0) |
| Gender (biological sex), n (%) | | | | | | | | | | | |
| Male | 1 (100) | 40 (85.1) | 58 (65.9) | 65 (69.9) | 4 (50) | 26 (89.7) | 78 (71.6) | 167 (82.3) | 53 (70.7) | 8 (50) | 500 (74.7) |
| Female | 0 (0) | 7 (14.9) | 30 (34.1) | 28 (30.1) | 4 (50) | 3 (10.3) | 31 (28.4) | 36 (17.7) | 22 (29.3) | 8 (50) | 169 (25.3) |
| Missing | 0 (0) | 0 (0) | 0 (0) | 0 (0) | 0 (0%) | 0 (0) | 0 (0) | 0 (0) | 0 (0) | 0 (0) | 0 (0) |
| Race, n (%) | | | | | | | | | | | |
| Asian | 0 (0) | 1 (2.1) | 4 (4.6) | 0 (0) | 0 (0) | 0 (0) | 109 (100) | 202 (99.5) | 75 (100) | 16 (100) | 407 (60.8) |
| White | 1 (100) | 45 (95.7) | 76 (86.4) | 93 (100) | 8 (100) | 28 (96.6) | 0 (0) | 0 (0) | 0 (0) | 0 (0) | 251 (37.5) |
| Black or African American | 0 (0) | 1 (2.1) | 2 (2.27) | 0 (0) | 0 (0) | 1 (3.45) | 0 (0) | 0 (0) | 0 (0) | 0 (0) | 4 (0.6) |
| Other | 0 (0) | 0 (0) | 0 (0) | 0 (0) | 0 (0) | 0 (0) | 0 (0) | 1 (0.5) | 0 (0) | 0 (0) | 1 (0.1) |
| Missing | 0 (0) | 0 (0) | 6 (6.8) | 0 (0) | 0 (0) | 0 (0) | 0 (0) | 0 (0) | 0 (0) | 0 (0) | 6 (0.9) |
| Ethnicity, n (%) | | | | | | | | | | | |
| Hispanic or Latino | 0 (0) | 1 (2.1) | 0 (0) | 0 (0) | 0 (0) | 0 (0) | 0 (0) | 0 (0) | 0 (0) | 0 (0) | 1 (0.1) |
| Not Hispanic or Latino | 1 (100) | 46 (97.9) | 82 (93.2) | 93 (100) | 8 (100) | 28 (96.6) | 109 (100) | 203 (100) | 75 (100) | 16 (100) | 661 (98.8) |
| Unknown | 0 (0) | 0 (0) | 6 (6.8) | 0 (0) | 0 (0) | 1 (3.5) | 0 (0) | 0 (0) | 0 (0) | 0 (0) | 7 (1.1) |
| Missing | 0 (0) | 0 (0) | 0 (0) | 0 (0) | 0 (0) | 0 (0) | 0 (0) | 0 (0) | 0 (0) | 0 (0) | 0 (0) |
| Smoking history, n (%) | | | | | | | | | | | |
| Never | 0 (0) | 11 (23.4) | 18 (20.5) | 35 (37.6) | 1 (12.5) | 4 (13.8) | 65 (59.6) | 122 (60.1) | 38 (50.7) | 10 (62.5) | 304 (45.4) |
| Former | 1 (100) | 16 (34) | 18 (20.5) | 32 (34.4) | 3 (37.5) | 4 (13.8) | 23 (21.1) | 35 (17.2) | 24 (32) | 1 (6.3) | 157 (23.5) |
| Current | 0 (0) | 13 (27.7) | 27 (30.7) | 19 (20.4) | 2 (25) | 8 (27.6) | 17 (15.6) | 43 (21.2) | 10 (13.3) | 4 (25) | 143 (21.4) |
| No Information | 0 (0) | 7 (14.9) | 25 (28.4) | 7 (7.5) | 2 (25) | 13 (44.8) | 4 (3.67) | 3 (1.5) | 3 (4) | 1 (6.3) | 65 (9.7) |
| Missing | 0 (0) | 0 (0) | 0 (0) | 0 (0) | 0 (0) | 0 (0) | 0 (0) | 0 (0) | 0 (0) | 0 (0) | 0 (0) |
| Ongoing antiviral therapy, n (%) | | | | | | | | | | | |
| Yes | 1 (100) | 4 (8.5) | 24 (27.3) | 2 (2.2) | 1 (12.5) | 4 (13.8) | 62 (56.9) | 132 (65) | 27 (36) | 4 (25) | 261 (39) |
| No | 0 (0) | 43 (91.5) | 64 (72.7) | 91 (97.8) | 7 (87.5) | 25 (86.2) | 47 (43.1) | 71 (35) | 48 (64) | 12 (75) | 408 (61) |
| Missing | 0 (0) | 0 (0) | 0 (0) | 0 (0) | 0 (0) | 0 (0) | 0 (0) | 0 (0) | 0 (0) | 0 (0) | 0 (0) |
| Antibiotics, n (%) | | | | | | | | | | | |
| Yes | 0 (0) | 2 (4.3) | 5 (5.7) | 10 (10.8) | 0 (0) | 2 (6.9) | 1 (0.9) | 1 (0.5) | 0 (0) | 0 (0) | 21 (3.1) |
| No | 1 (100) | 45 (95.7) | 83 (94.3) | 83 (89.2) | 8 (100) | 27 (93.1) | 108 (99.1) | 202 (99.5) | 75 (100) | 16 (100) | 648 (96.9) |
| Missing | 0 (0) | 0 (0) | 0 (0) | 0 (0) | 0 (0) | 0 (0) | 0 (0) | 0 (0) | 0 (0) | 0 (0) | 0 (0) |
| Group, n (%) | | | | | | | | | | | |
| HCC case | 1 (100) | 38 (80.9) | 39 (44.3) | 41 (44.1) | 2 (25) | 21 (72.4) | 56 (51.4) | 120 (59.1) | 40 (53.3) | 8 (50) | 366 (54.7) |
| Control | 0 (0) | 9 (19.1) | 49 (55.7) | 52 (55.9) | 6 (75) | 8 (27.6) | 53 48.6) | 83 (40.9) | 35 (46.7) | 8 (50) | 303 (45.3) |
| Missing | 0 (0) | 0 (0) | 0 (0) | 0 (0) | 0 (0) | 0 (0) | 0 (0) | 0 (0) | 0 (0) | 0 (0) | 0 (0) |

***Abbreviations:*** *SD, standard deviation; HCC, hepatocellular carcinoma.*

**Supplementary Table 2. Clinical performance of AFP, PIVKA-II and GAAD for detection of early-, late- and all-stage HCC at the pre-defined cut-offs: Clinical validation cohort**

| Parameter | AFP | | | PIVKA-II | | | GAAD | | |
| --- | --- | --- | --- | --- | --- | --- | --- | --- | --- |
| Staging | Early-stage | Late-stage | All-stage | Early-stage | Late-stage | All-stage | Early-stage | Late-stage | All-stage |
| N (HCC/control) | 477 (174/303) | 495 (192/303) | 669 (366/303) | 477 (174/303) | 495 (192/303) | 669 (366/303) | 477 (174/303) | 495 (192/303) | 669 (366/303) |
| True positives | 72 | 125 | 197 | 106 | 181 | 287 | 122 | 182 | 304 |
| True negatives | 297 | 297 | 297 | 274 | 274 | 274 | 284 | 284 | 284 |
| False positives | 6 | 6 | 6 | 29 | 29 | 29 | 19 | 19 | 19 |
| False negatives | 102 | 67 | 169 | 68 | 11 | 79 | 52 | 10 | 62 |
| Sensitivity  (95% Cl) | 41.4  (34–49.1) | 65.1  (57.9–71.8) | 53.8  (48.6–59) | 60.9  (53.2–68.2) | 94.3  (90–97.1) | 78.4  (73.8–82.5) | 70.1  (62.7–76.8) | 94.8  (90.6–97.5) | 83.1  (78.8–86.8) |
| Specificity  (95% Cl) | 98.0  (95.7–99.3) | 98.0 (95.7–99.3) | 98.0 (95.7–99.3) | 90.4  (86.5–93.5) | 90.4  (86.5–93.5) | 90.4  (86.5–93.5) | 93.7  (90.4–96.2) | 93.7 (90.4–96.2) | 93.7  (90.4–96.2) |
| PPV 1% prev | 17.4 | 24.9 | 21.5 | 6.04 | 9.05 | 7.6 | 10.1 | 13.2 | 11.8 |
| PPV 2% prev | 29.9 | 40.2 | 35.7 | 11.5 | 16.7 | 14.3 | 18.6 | 23.6 | 21.3 |
| PPV 3% prev | 39.3 | 50.4 | 45.7 | 16.4 | 23.3 | 20.2 | 25.7 | 31.9 | 29.1 |
| PPV 4% prev | 46.5 | 57.8 | 53.1 | 21 | 29.1 | 25.4 | 31.8 | 38.6 | 35.6 |
| PPV 5% prev | 52.4 | 63.4 | 58.9 | 25.1 | 34.1 | 30.1 | 37 | 44.3 | 41.1 |
| NPV 1% prev | 99.4 | 99.6 | 99.5 | 99.6 | 99.9 | 99.8 | 99.7 | 99.9 | 99.8 |
| NPV 2% prev | 98.8 | 99.3 | 99 | 99.1 | 99.9 | 99.5 | 99.4 | 99.9 | 99.6 |
| NPV 3% prev | 98.2 | 98.9 | 98.6 | 98.7 | 99.8 | 99.3 | 99 | 99.8 | 99.4 |
| NPV 4% prev | 97.6 | 98.5 | 98.1 | 98.2 | 99.7 | 99 | 98.7 | 99.8 | 99.3 |
| NPV 5% prev | 96.9 | 98.2 | 97.6 | 97.8 | 99.7 | 98.8 | 98.3 | 99.7 | 99.1 |

# *Abbreviations: AFP, alpha-fetoprotein; CI, confidence interval; DCP, des-gamma carboxy-prothrombin; GAAD, Gender (biological sex), Age, AFP, DCP; HCC, hepatocellular carcinoma; NPV, negative predictive value; PIVKA-II, protein induced by vitamin K absence-II; PPV, positive predictive value.*

**Supplementary Table 3. Cut-offs of Elecsys AFP, Elecsys PIVKA-II and GAAD at specified sensitivity: Clinical validation cohort**

| **AFP** | | | | | | | | | | | | | | | |
| --- | --- | --- | --- | --- | --- | --- | --- | --- | --- | --- | --- | --- | --- | --- | --- |
|  | **AFP** | **Sensitivity all-stage** | **Sensitivity early-stage** | **Sensitivity late-stage** | **Specificity** | **PPV 1%** | **PPV 2%** | **PPV 3%** | **PPV 4%** | **PPV 5%** | **NPV 1%** | **NPV 2%** | **NPV 3%** | **NPV 4%** | **NPV 5%** |
| Sensitivity 70% | 6.94 | 70.2  (65.2–74.9) | 59.2  (51.5–66.6) | 80.2  (73.9–85.6) | 86.8  (82.5–90.4) | 5.1 | 9.8 | 14.1 | 18.1 | 21.9 | 99.7 | 99.3 | 98.9 | 98.6 | 98.2 |
| Sensitivity 75% | 5.88 | 75.1  (70.4–79.5) | 65.5  (57.9–72.5) | 83.9  (77.9–88.8) | 83.5  (78.8–87.5) | 4.4 | 8.5 | 12.3 | 15.9 | 19.3 | 99.7 | 99.4 | 99.1 | 98.8 | 98.5 |
| Sensitivity 80% | 5.03 | 80.1  (75.6–84) | 71.8  (64.5–78.4) | 87.5  (82–91.8) | 76.9  (71.7–81.5) | 3.4 | 6.6 | 9.7 | 12.6 | 15.4 | 99.7 | 99.5 | 99.2 | 98.9 | 98.7 |
| Sensitivity 85% | 4.23 | 85.2  (81.2–88.7) | 78.2  (71.3–84.1) | 91.7  (86.8–95.2) | 70.3  (64.8–75.4) | 2.8 | 5.5 | 8.2 | 10.7 | 13.1 | 99.8 | 99.6 | 99.4 | 99.1 | 98.9 |
| Sensitivity 90% | 3.62 | 90.2  (86.6–93) | 84.5  (78.2–89.5) | 95.3  (91.3–97.8) | 63.4  (57.7–68.8) | 2.4 | 4.8 | 7.1 | 9.3 | 11.5 | 99.8 | 99.7 | 99.5 | 99.4 | 99.2 |
| Sensitivity 95% | 2.21 | 95.1  (92.3–97.1) | 92.5  (87.6–96) | 97.4  (94–99.1) | 26.4  (21.5–31.7) | 1.3 | 2.6 | 3.8 | 5.1 | 6.4 | 99.8 | 99.6 | 99.4 | 99.2 | 99 |
| **PIVKA-II** | | | | | | | | | | | | | | | |
|  | **PIVKA-II** | **Sensitivity all-stage** | **Sensitivity early-stage** | **Sensitivity late-stage** | **Specificity** | **PPV 1%** | **PPV 2%** | **PPV 3%** | **PPV 4%** | **PPV 5%** | **NPV 1%** | **NPV 2%** | **NPV 3%** | **NPV 4%** | **NPV 5%** |
| Sensitivity 70% | 47.2 | 69.9  (65–74.6) | 46.6  (39–54.3) | 91.1  (86.2–94.8) | 93.7  (90.4–96.2) | 10.1 | 18.5 | 25.6 | 31.7 | 37 | 99.7 | 99.3 | 99 | 98.7 | 98.3 |
| Sensitivity 75% | 33.8 | 75.1  (70.4–79.5) | 55.7  (48–63.3) | 92.7  (88.1–96) | 92.4  (88.8–95.1) | 9.1 | 16.8 | 23.4 | 29.2 | 34.3 | 99.7 | 99.5 | 99.2 | 98.9 | 98.6 |
| Sensitivity 80% | 27.8 | 80.1  (75.6–84) | 63.8  (56.2–70.9) | 94.8  (90.6–97.5) | 90.1  (86.2–93.2) | 7.6 | 14.2 | 20 | 25.2 | 29.9 | 99.8 | 99.6 | 99.3 | 99.1 | 98.8 |
| Sensitivity 85% | 19.3 | 85.2  (81.2–88.7) | 73  (65.7–79.4) | 96.4  (92.6–98.5) | 71.6  (66.2–76.6) | 2.9 | 5.8 | 8.5 | 11.1 | 13.6 | 99.8 | 99.6 | 99.4 | 99.1 | 98.9 |
| Sensitivity 90% | 16.7 | 89.3  (85.7–92.3) | 79.3  (72.5–85.1) | 98.4  (95.5–99.7) | 45.9  (40.2–51.7) | 1.6 | 3.3 | 4.9 | 6.4 | 8 | 99.8 | 99.5 | 99.3 | 99 | 98.8 |
| Sensitivity 95% | 14 | 95.4  (92.7–97.3) | 90.2  (84.8–94.2) | 100  (98.1–100) | 19.5  (15.2–24.4) | 1.2 | 2.4 | 3.5 | 4.7 | 5.9 | 99.8 | 99.5 | 99.3 | 99 | 98.8 |
| **GAAD** | | | | | | | | | | | | | | | |
|  | **GAAD** | **Sensitivity all-stage** | **Sensitivity early-stage** | **Sensitivity late-stage** | **Specificity** | **PPV 1%** | **PPV 2%** | **PPV 3%** | **PPV 4%** | **PPV 5%** | **NPV 1%** | **NPV 2%** | **NPV 3%** | **NPV 4%** | **NPV 5%** |
| Sensitivity 70% | 6.12 | 70.2  (65.2–74.9) | 50.6  (42.9–58.2) | 88  (82.6–92.3) | 98.7  (96.7–99.6) | 34.9 | 52.1 | 62.2 | 68.9 | 73.7 | 99.7 | 99.4 | 99.1 | 98.8 | 98.4 |
| Sensitivity 75% | 4.46 | 75.1  (70.4–79.5) | 57.5  (49.8–64.9) | 91.1  (86.2–94.8) | 98  (95.7–99.3) | 27.7 | 43.6 | 54 | 61.3 | 66.6 | 99.7 | 99.5 | 99.2 | 99 | 98.7 |
| Sensitivity 80% | 3.45 | 80.1  (75.6–84) | 63.8  (56.2–70.9) | 94.8  (90.6–97.5) | 96  (93.2–97.9) | 17 | 29.2 | 38.5 | 45.7 | 51.5 | 99.8 | 99.6 | 99.4 | 99.1 | 98.9 |
| Sensitivity 85% | 1.88 | 85  (80.9–88.5) | 73  (65.7–79.4) | 95.8  (92–98.2) | 89.4  (85.4–92.7) | 7.5 | 14.1 | 19.9 | 25.1 | 29.7 | 99.8 | 99.7 | 99.5 | 99.3 | 99.1 |
| Sensitivity 90% | 1.15 | 89.9  (86.3–92.8) | 81.6  (75–87.1) | 97.4  (94–99.1) | 84.8  (80.3–88.7) | 5.6 | 10.8 | 15.5 | 19.8 | 23.8 | 99.9 | 99.8 | 99.6 | 99.5 | 99.4 |
| Sensitivity 95% | 0.68 | 94.8  (92–96.8) | 90.8  (85.5–94.7) | 98.4  (95.5–99.7) | 71.6 (66.2–76.6) | 3.3 | 6.4 | 9.4 | 12.2 | 15 | 99.9 | 99.9 | 99.8 | 99.7 | 99.6 |

***Abbreviations:*** *AFP, alpha -fetoprotein; DCP, des-gamma carboxy-prothrombin; GAAD, Gender (biological sex), Age, AFP, DCP; HCC, hepatocellular carcinoma; NPV, negative predictive value; PIVKA-II, protein induced by vitamin K absence-II; PPV, positive predictive value.*

**Supplementary Table 4. Cut-offs of Elecsys AFP, Elecsys PIVKA-II and GAAD at specified specificity: Clinical Validation Cohort**

| **AFP** | | | | | | | | | | | | | | | |
| --- | --- | --- | --- | --- | --- | --- | --- | --- | --- | --- | --- | --- | --- | --- | --- |
|  | **AFP** | **Sensitivity all-stage** | **Sensitivity early-stage** | **Sensitivity late-stage** | **Specificity** | **PPV 1%** | **PPV 2%** | **PPV 3%** | **PPV 4%** | **PPV 5%** | **NPV 1%** | **NPV 2%** | **NPV 3%** | **NPV 4%** | **NPV 5%** |
| Specificity 70% | 4.17 | 85.5  (81.5–89) | 78.2  (71.3–84.1) | 92.2  (87.4–95.6) | 70  (64.5–75.1) | 2.8 | 5.5 | 8.1 | 10.6 | 13 | 99.8 | 99.6 | 99.4 | 99.1 | 98.9 |
| Specificity 75% | 4.69 | 81.4  (77.1–85.3) | 73.6  (66.4–79.9) | 88.5  (83.2–92.7) | 74.9 (69.6–79.7) | 3.2 | 6.2 | 9.1 | 11.9 | 14.6 | 99.8 | 99.5 | 99.2 | 99 | 98.7 |
| Specificity 80% | 5.33 | 78.4  (73.8–82.5) | 69  (61.5–75.7) | 87  (81.4–91.4) | 79.5  (74.6–83.9) | 3.7 | 7.3 | 10.6 | 13.8 | 16.8 | 99.7 | 99.4 | 99.2 | 98.9 | 98.6 |
| Specificity 85% | 6.09 | 73 (68.1–77.4) | 61.5  (53.8–68.8) | 83.3  (77.3–88.3) | 84.8  (80.3–88.7) | 4.6 | 8.9 | 12.9 | 16.7 | 20.2 | 99.7 | 99.4 | 99 | 98.7 | 98.3 |
| Specificity 90% | 8.51 | 67.2  (62.1–72) | 58  (50.3–65.5) | 75.5  (68.8–81.4) | 89.8  (85.8–92.9) | 6.2 | 11.8 | 16.9 | 21.5 | 25.7 | 99.6 | 99.3 | 98.9 | 98.5 | 98.1 |
| Specificity 95% | 12.8 | 60.4  (55.2–65.4) | 49.4  (41.8–57.1) | 70.3  (63.3–76.7) | 94.7  (91.6–97) | 10.4 | 18.9 | 26.1 | 32.3 | 37.6 | 99.6 | 99.2 | 98.7 | 98.3 | 97.8 |
| **PIVKA-II** | | | | | | | | | | | | | | | |
|  | **PIVKA-II** | **Sensitivity all-stage** | **Sensitivity early-stage** | **Sensitivity late-stage** | **Specificity** | **PPV 1%** | **PPV 2%** | **PPV 3%** | **PPV 4%** | **PPV 5%** | **NPV 1%** | **NPV 2%** | **NPV 3%** | **NPV 4%** | **NPV 5%** |
| Specificity 70% | 19.1 | 85.5  (81.5–89) | 73  (65.7–79.4) | 96.9  (93.3–98.8) | 70  (64.5–75.1) | 2.8 | 5.5 | 8.1 | 10.6 | 13 | 99.8 | 99.6 | 99.4 | 99.1 | 98.9 |
| Specificity 75% | 20.2 | 85  (80.9–88.5) | 72.4  (65.1–78.9) | 96.4  (92.6–98.5) | 74.9  (69.6–79.7) | 3.3 | 6.5 | 9.5 | 12.4 | 15.1 | 99.8 | 99.6 | 99.4 | 99.2 | 99 |
| Specificity 80% | 21.7 | 83.9  (79.7–87.5) | 70.7  (63.3–77.3) | 95.8  (92–98.2) | 80.2  (75.3–84.5) | 4.1 | 8 | 11.6 | 15 | 18.2 | 99.8 | 99.6 | 99.4 | 99.2 | 99 |
| Specificity 85% | 23.4 | 82.2  (77.9–86) | 68.4  (60.9–75.2) | 94.8  (90.6–97.5) | 85.1  (80.6–89) | 5.3 | 10.2 | 14.6 | 18.7 | 22.6 | 99.8 | 99.6 | 99.4 | 99.1 | 98.9 |
| Specificity 90% | 27.5 | 80.3  (75.9–84.3) | 64.4  (56.8–71.5) | 94.8  (90.6–97.5) | 89.8  (85.8–92.9) | 7.4 | 13.8 | 19.5 | 24.7 | 29.2 | 99.8 | 99.6 | 99.3 | 99.1 | 98.9 |
| Specificity 95% | 58.9 | 67.5  (62.4–72.3) | 42  (34.5–49.7) | 90.6  (85.6–94.3) | 95  (92–97.2) | 12.1 | 21.8 | 29.7 | 36.2 | 41.8 | 99.7 | 99.3 | 99 | 98.6 | 98.2 |
| **GAAD** | | | | | | | | | | | | | | | |
|  | **GAAD** | **Sensitivity all-stage** | **Sensitivity early-stage** | **Sensitivity late-stage** | **Specificity** | **PPV 1%** | **PPV 2%** | **PPV 3%** | **PPV 4%** | **PPV 5%** | **NPV 1%** | **NPV 2%** | **NPV 3%** | **NPV 4%** | **NPV 5%** |
| Specificity 70% | 0.65 | 95.6  (93–97.5) | 92.5  (87.6–96) | 98.4  (95.5–99.7) | 70.3  (64.8–75.4) | 3.2 | 6.2 | 9.1 | 11.8 | 14.5 | 99.9 | 99.9 | 99.8 | 99.7 | 99.7 |
| Specificity 75% | 0.78 | 93.7  (90.7–96) | 88.5  (82.8–92.8) | 98.4  (95.5–99.7) | 74.9  (69.6–79.7) | 3.6 | 7.1 | 10.4 | 13.5 | 16.4 | 99.9 | 99.8 | 99.7 | 99.7 | 99.6 |
| Specificity 80% | 1.01 | 90.7  (87.3–93.5) | 82.8  (76.3–88.1) | 97.9  (94.8–99.4) | 79.9  (74.9–84.2) | 4.4 | 8.4 | 12.2 | 15.8 | 19.2 | 99.9 | 99.8 | 99.6 | 99.5 | 99.4 |
| Specificity 85% | 1.21 | 89.6  (86–92.5) | 81  (74.4–86.6) | 97.4  (94–99.1) | 85.1  (80.6–89) | 5.8 | 11 | 15.7 | 20.1 | 24.1 | 99.9 | 99.8 | 99.6 | 99.5 | 99.4 |
| Specificity 90% | 2 | 84.7  (80.6–88.2) | 72.4  (65.1–78.9) | 95.8  (92–98.2) | 89.8  (85.8–92.9) | 7.7 | 14.5 | 20.4 | 25.6 | 30.3 | 99.8 | 99.7 | 99.5 | 99.3 | 99.1 |
| Specificity 95% | 2.83 | 82.5  (78.2–86.3) | 69  (61.5–75.7) | 94.8  (90.6–97.5) | 94.7  (91.6–97) | 13.6 | 24.2 | 32.6 | 39.4 | 45.1 | 99.8 | 99.6 | 99.4 | 99.2 | 99 |

***Abbreviations:*** *AFP, alpha -fetoprotein; DCP, des-gamma carboxy-prothrombin; GAAD, Gender (biological sex), Age, AFP, DCP; HCC, hepatocellular carcinoma; NPV, negative predictive value; PIVKA-II, protein induced by vitamin K absence-II; PPV, positive predictive value.*

**Supplementary Table 5. Clinical performance of AFP, PIVKA-II and GAAD for detection of early-, late- and all-stage HCC at the pre-defined cut-offs: Algorithm development cohort**

| Parameter | AFP | | | PIVKA-II | | | GAAD | | |
| --- | --- | --- | --- | --- | --- | --- | --- | --- | --- |
| Staging | Early-stage | Late-stage | All-stage | Early-stage | Late-stage | All-stage | Early-stage | Late-stage | All-stage |
| N (HCC/control) | 855 (125/730) | 896 (166/730) | 1032 (302/730) | 851 (124/727) | 892 (165/727) | 1027 (300/727) | 851 (124/727) | 892 (165/727) | 1027 (300/727) |
| True positives | 53 | 121 | 181 | 76 | 154 | 240 | 89 | 157 | 256 |
| True negatives | 693 | 693 | 693 | 645 | 645 | 645 | 654 | 654 | 654 |
| False positives | 37 | 37 | 37 | 82 | 82 | 82 | 73 | 73 | 73 |
| False negatives | 72 | 45 | 121 | 48 | 11 | 60 | 35 | 8 | 44 |
| Sensitivity  (95% Cl) | 42.4  (33.6–51.6) | 72.9  (65.5–79.5) | 59.9  (54.2–65.5) | 61.3  (52.1–69.9) | 93.3  (88.4–96.6) | 80  (75–84.4) | 71.8  (63–79.5) | 95.2(90.7–97.9) | 85.3  (80.8–89.1) |
| Specificity  (95% Cl) | 94.9  (93.1–96.4) | 94.9  (93.1–96.4) | 94.9  (93.1–96.4) | 88.7  (86.2–90.9) | 88.7  (86.2–90.9) | 88.7  (86.2–90.9) | 90  (87.5–92) | 90 (87.5–92) | 90  (87.5–92) |
| PPV 1% prev | 7.8 | 12.7 | 10.7 | 5.2 | 7.7 | 6.7 | 6.7 | 8.7 | 7.9 |
| PPV 2% prev | 14.6 | 22.7 | 19.4 | 10.0 | 14.4 | 12.6 | 12.7 | 16.2 | 14.8 |
| PPV 3% prev | 20.6 | 30.8 | 26.8 | 14.4 | 20.4 | 18 | 18.1 | 22.7 | 20.8 |
| PPV 4% prev | 25.8 | 37.5 | 33 | 18.5 | 25.6 | 22.8 | 22.9 | 28.3 | 26.1 |
| PPV 5% prev | 30.6 | 43.1 | 38.4 | 22.2 | 30.3 | 27.2 | 27.3 | 33.3 | 30.9 |
| NPV 1% prev | 99.4 | 99.7 | 99.6 | 99.6 | 99.9 | 99.8 | 99.7 | 99.9 | 99.8 |
| NPV 2% prev | 98.8 | 99.4 | 99.1 | 99.1 | 99.8 | 99.5 | 99.4 | 99.9 | 99.7 |
| NPV 3% prev | 98.2 | 99.1 | 98.7 | 98.7 | 99.8 | 99.3 | 99 | 99.8 | 99.5 |
| NPV 4% prev | 97.5 | 98.8 | 98.3 | 98.2 | 99.7 | 99.1 | 98.7 | 99.8 | 99.3 |
| NPV 5% prev | 96.9 | 98.5 | 97.8 | 97.8 | 99.6 | 98.8 | 98.4 | 99.7 | 99.1 |

*Abbreviations: AFP, alpha-fetoprotein; CI, confidence interval; DCP, des-gamma carboxy-prothrombin; GAAD, Gender (biological sex), Age, AFP, DCP; HCC, hepatocellular carcinoma; NPV, negative predictive value; PIVKA-II, protein induced by vitamin K absence-II; PPV, positive predictive value.*

# Supplementary Figure 1. The GAAD score ranges across HCC disease stages and in benign disease controls.

**
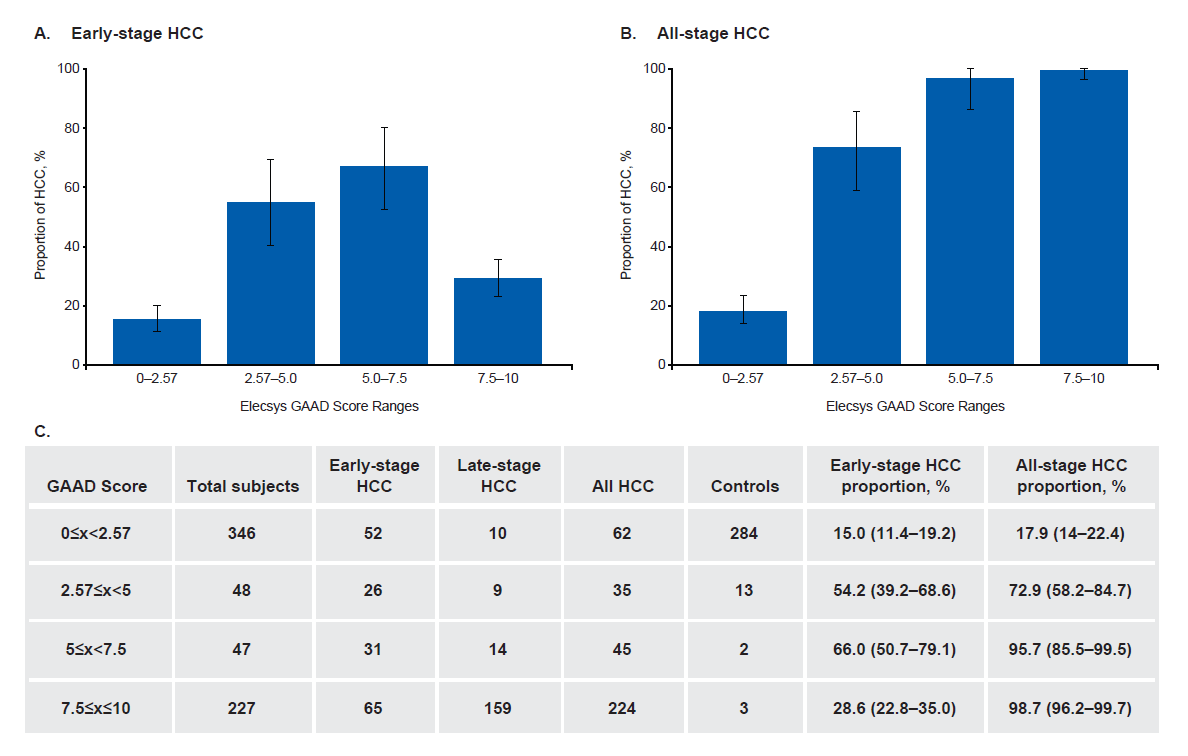
**

***Abbreviations:*** *AFP, alpha-fetoprotein; DCP, des-gamma carboxy-prothrombin; GAAD, Gender (biological sex), Age, AFP, DCP;* *HCC, hepatocellular carcinoma.*

**Supplementary Figure 2. Distribution of GAAD, AFP and PIVKA-II scores across (A) HCC cases and benign disease controls; (B) according to BCLC disease stage.**

**
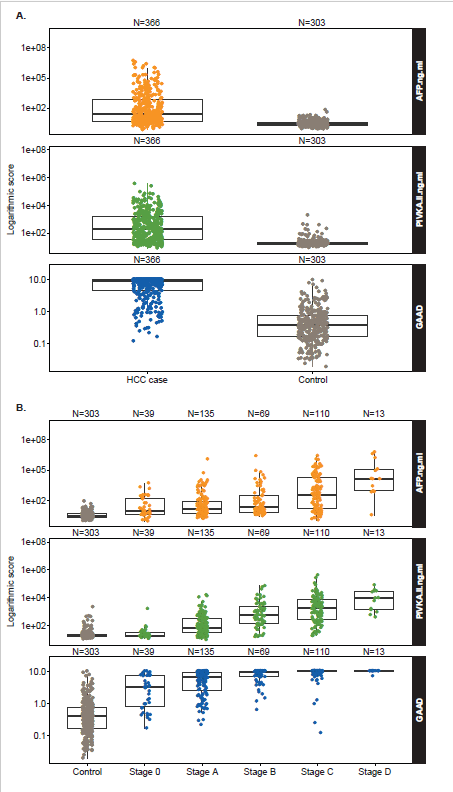
**

***Abbreviations:*** *AFP, alpha-fetoprotein; BCLC, Barcelona Clinic Liver Cancer; DCP, des-gamma carboxy-prothrombin; GAAD, Gender (biological sex), Age, AFP, DCP; PIVKA-II, protein induced by vitamin K absence-II.*

**Supplementary Figure 3. Distribution of GAAD, AFP and PIVKA-II scores (A) according to disease etiology within HCC cases and benign disease controls; (B) according to region.**


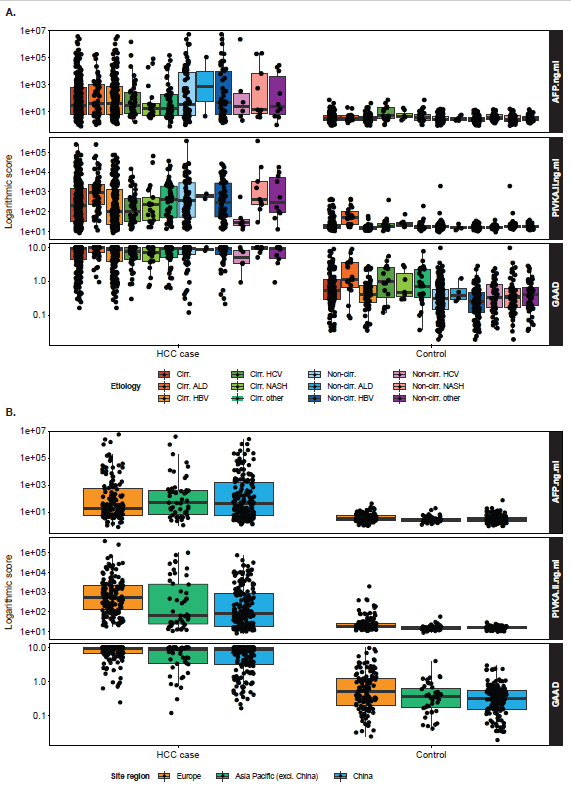


***Abbreviations:*** *ALD, alcoholic liver disease; AFP, alpha-fetoprotein; BCLC, Barcelona Clinic Liver Cancer; DCP, des-gamma carboxy-prothrombin; GAAD, Gender (biological sex), Age, AFP, DCP; HBV, hepatitis B virus; HCC, hepatocellular carcinoma; HCV, hepatitis C virus; NASH, non-alcoholic steatohepatitis; PIVKA-II, protein induced by vitamin K absence-II.*

**Supplementary Figure 4. Venn diagrams to illustrate the number of early cases, late cases, all cases, or controls detected by the single markers (Elecsys AFP, Elecsys PIVKA-II) and the GAAD algorithm using the predefined cutoffs**

**
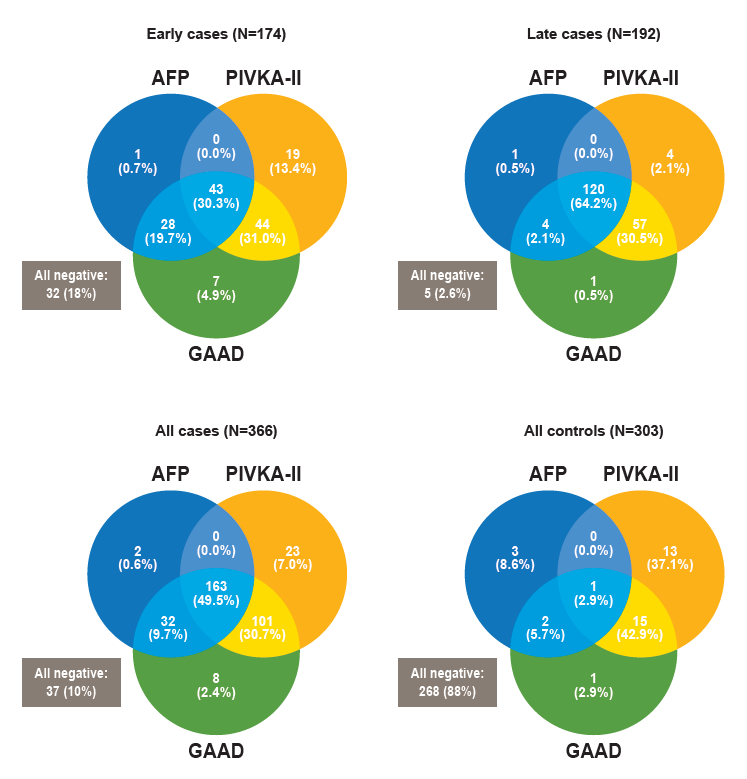
**

***Abbreviations:*** *AFP, alpha-fetoprotein; DCP, des-gamma carboxy-prothrombin; GAAD, Gender (biological sex), Age, AFP, DCP; PIVKA-II, protein induced by vitamin K absence-II.*

**Supplementary Figure 5. Simulations of the different age distributions between cases and controls and the impact on the clinical performance of GAAD: Algorithm development cohort**

**
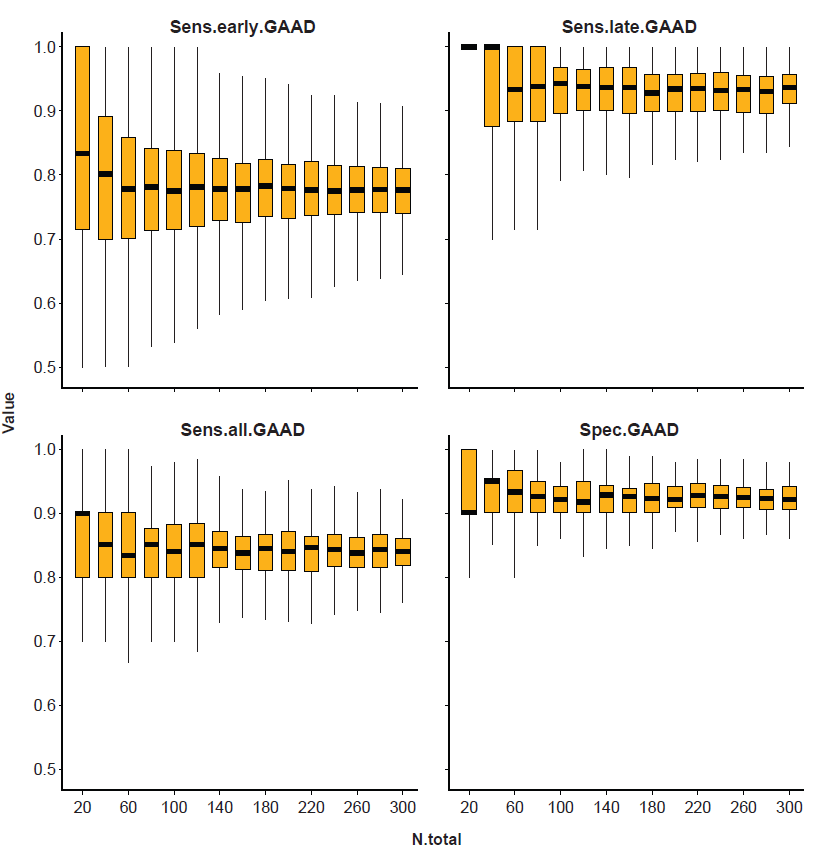
**

***Abbreviations:*** *AFP, alpha-fetoprotein; DCP, des-gamma carboxy-prothrombin; GAAD, Gender (biological sex), Age, AFP, DCP.*

**Supplementary Figure 6. Simulations of the different gender distributions between cases and controls and the impact on the clinical performance of GAAD: Algorithm development cohort**

***
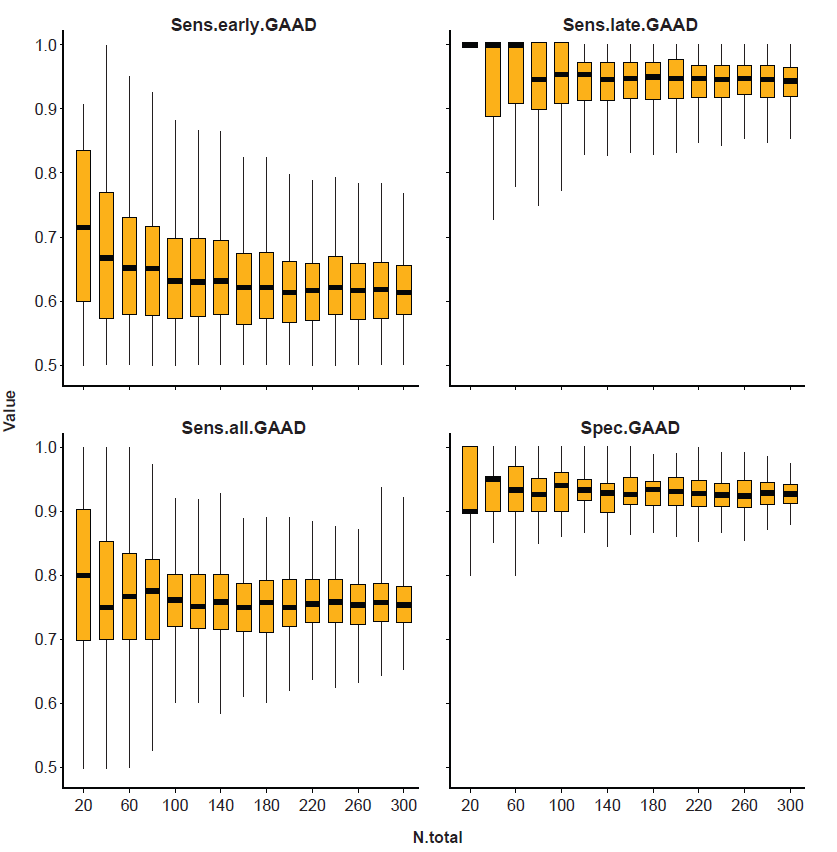
***

***Abbreviations:*** *AFP, alpha-fetoprotein; DCP, des-gamma carboxy-prothrombin; GAAD, Gender (biological sex), Age, AFP, DCP.*

**Supplementary Figure 7. Clinical performance of GAAD, GALAD, AFP, AFP-L3 and PIVKA-II for the detection of (A) early-stage HCC, (B) all-stage HCC, (C) cirrhotic early-stage HCC and (D) non-cirrhotic early-stage HCC: Subanalysis of the clinical validation cohort**

**
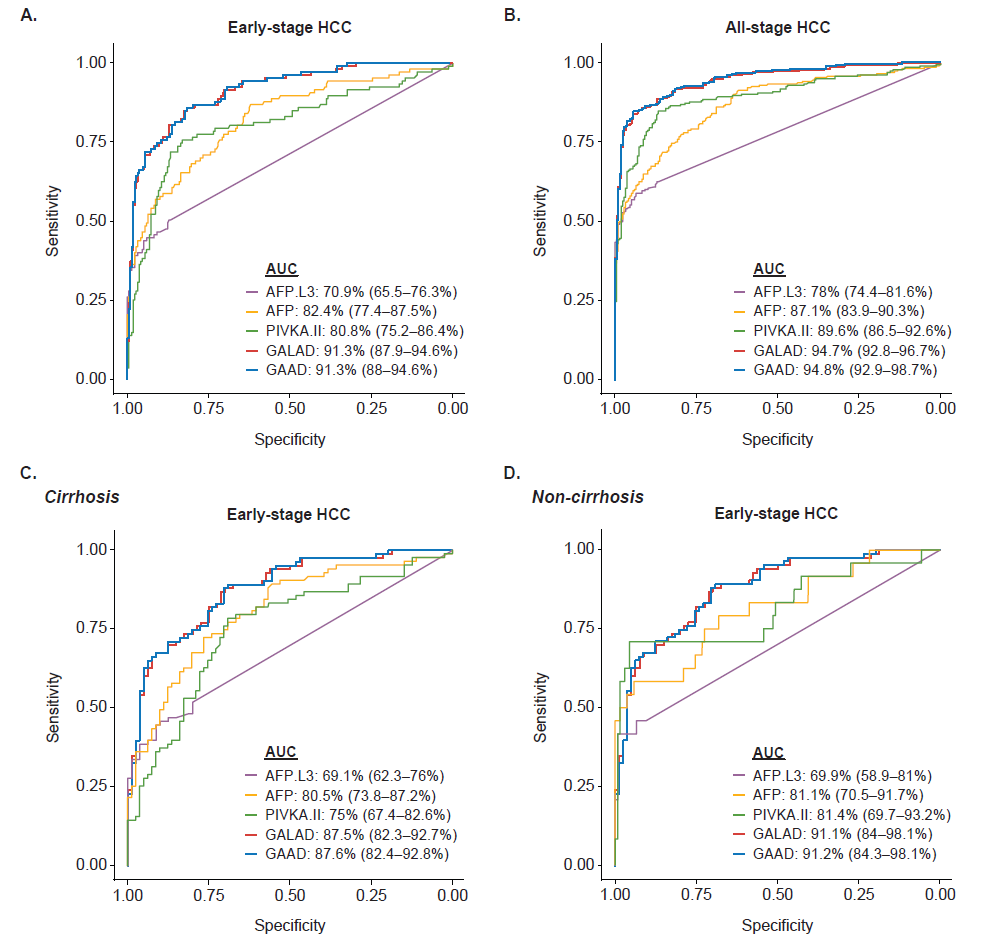
**

***Abbreviations:*** *AFP, alpha-fetoprotein; AFP-L3, AFP isoform L3; AUC, area under the curve; DCP, des-gamma carboxy-prothrombin; HCC, hepatocellular carcinoma; GAAD, Gender (biological sex), Age, AFP, DCP; GALAD, Gender (biological sex), Age, AFP-L3, AFP, DCP; PIVKA-II, protein induced by vitamin K absence-II.*
